# Immunotherapy for neuromyelitis optica spectrum disorder: a comparative analysis of efficacy and safety of azathioprine, mycophenolate mofetil, tacrolimus, and rituximab

**DOI:** 10.3389/fneur.2025.1559118

**Published:** 2025-04-28

**Authors:** Jing Zhou, Xiaolin Yang, Xinyi Wang, Bin Li, Yun Xu, Haoran Zhang, Yinxin Zhu, Xiaoming Wang, Jinzhou Feng

**Affiliations:** ^1^Jinan University, Guangzhou, Guangdong, China; ^2^Department of Neurology, Affiliated Hospital of North Sichuan Medical College, Nanchong, Sichuan, China; ^3^Department of Neurology, First Affiliated Hospital of Chongqing Medical University, Chongqing, China; ^4^North Sichuan Medical College, Nanchong, China; ^5^First Affiliated Hospital of Chongqing Medical University, Chongqing, China; ^6^Beijing Tiantan Hospital, Capital Medical University, Beijing, China

**Keywords:** neuromyelitis optica spectrum disorder, off-label, traditional immunosuppressants, rituximab, economy

## Abstract

**Background and purpose:**

Biologic therapies are anticipated to dominate the treatment landscape for neuromyelitis optica spectrum disorders (NMOSD) in the future. Despite this, many patients in China continue to use off-label medications due to economic and other constraints. A multicenter NMOSD cohort study was conducted to compare the efficacy and safety of tacrolimus (TAC), mycophenolate mofetil (MMF), azathioprine (AZA), and rituximab (RTX). The objective of this study is to provide a clinical evidence-based reference for patients who still require the use of these off-label medications.

**Methods:**

This retrospective study included NMOSD patients treated with TAC (*n* = 24), MMF (*n* = 74), AZA (*n* = 34), and RTX (*n* = 81). Of these, 81 underwent magnetic resonance imaging (MRI) activity analysis during follow-up. The observation period commenced with the treatment initiation and extended until August 31, 2023. The primary efficacy outcome was the time to the first relapse post-immunotherapy initiation, The hazard ratio (HR) was analyzed using the Cox proportional hazards model to compare the relative risk of the first relapse between different treatment groups (e.g., RTX, MMF, TAC, and AZA). Secondary outcomes encompassed annualized relapse rate (ARR), MRI activity, drug persistence, and relapse rate (RR). The safety outcome was the occurrence of severe adverse drug reaction events.

**Results:**

A total of 213 patients were included in the study. During the first year of immunotherapy, patients treated with RTX (HR = 18.41, 95% CI: 4.039–83.87; *p* < 0.05) and MMF (HR = 22.72, 95% CI: 4.783–108.0; *p* < 0.0001) experienced a significantly lower risk of relapse compared to those treated with tacrolimus (TAC). The risk of first relapse in the AZA group was higher compared to the RTX group (HR = 2.786, 95% CI: 0.4771–16.27; *p* = 0.2551) and the MMF group (HR = 4.005, 95% CI: 0.5973–26.86; *p* = 0.1529), although the differences were not statistically significant. In the second year, this trend continued with RTX (HR = 6.200, 95% CI: 1.825–21.06; *p* = 0.0034) and MMF (HR = 6.017, 95% CI: 1.782–20.32; *p* = 0.0039) demonstrating a lower relapse risk compared to oral TAC. Similarly, RTX and MMF were more effective than oral AZA in reducing relapse risk (RTX: HR = 3.510, 95% CI: 1.202–10.25; *p* = 0.0216; MMF: HR = 3.909, 95% CI: 1.318–11.59; *p* = 0.0140). The difference in the risk of the first relapse between the MMF and RTX groups was not statistically significant (HR = 0.7217, *p* = 0.7156 in the first year; HR = 0.9351, *p* = 0.9003 in the second year) although the difference was not statistically significant. The risk of first relapse was higher in the group treated with oral conventional immunosuppressants (ISTs) compared to the RTX group, (HR = 2.170, *p* = 0.1449 in the first year; HR = 1.820, *p* = 0.1091 in the second year). The annual relapse rate (ARR) significantly decreased after treatment with all four drugs. RTX and MMF were more effective in controlling disease relapse compared to TAC and AZA, though these differences were not statistically significant (RTX: ARR = 0.12, 95% CI: 0.03–0.21; MMF: ARR = 0.15, 95% CI: 0.07–0.23; TAC: ARR = 0.21, 95% CI: 0.03–0.39; AZA: ARR = 0.19, 95% CI: 0.08–0.3; *p* = 0.81). When combining clinical and relapse-independent MRI activity analyses in 81 NMOSD patients, RTX demonstrated superior control of disease activity, with a statistically significant difference (*p* = 0.036). No hospitalization events related to severe drug adverse effects were reported in either the IST or RTX groups.

**Conclusion:**

The study provides data comparing the efficacy of various off-label treatments in a Chinese NMOSD cohort, illustrating that RTX is more effective than traditional immunosuppressants in controlling NMOSD relapses and disease activity but no superiority in the time to the first relapse post-immunotherapy initiation. RTX and MMF may offer superior treatment alternatives for NMOSD patients compared to TAC and AZA.

## Introduction

Neuromyelitis optica spectrum disorder (NMOSD) is a rare, autoimmune-mediated, inflammatory demyelinating disease of the central nervous system. It is characterized by recurrent severe optic neuritis and longitudinally extensive transverse myelitis (1–3). Approximately one-third of cases occur in Asia and other non-Caucasian populations, with global incidence rates ranging from 0.5 to 10 per 100,000 person-years (4–6) and an incidence of about 0.278 per 100,000 person-years in China (7). The disability associated with NMOSD primarily results from poor recovery following relapses, with even a single episode potentially leading to severe and irreversible disability (8, 9). In the natural progression of NMOSD, 40–60% of patients experience a relapse within 1 year, 90% within 3 years, and about 50% develop severe visual or motor impairments within 5–10 years. Currently, no cure exists for NMOSD, and the Neuromyelitis Optica Study Group (NEMOS) recommends early initiation of immunotherapy post-diagnosis to prevent relapses (10).

Since 2004, pathophysiological research on NMOSD has identified multiple therapeutic targets, such as CD19, theinterleukin-6 (IL-6) pathway, and the complement pathway (11), facilitating a shift in NMOSD immunotherapy toward personalized precision medicine. Based on the results of five international multicenter randomized controlled trials, all therapies approved by the FDA (U.S. Food and Drug Administration) and EMA (European Medicines Agency) for AQP4-IgG (aquaporin-4 immunoglobulin G)-positive NMOSD are biologics, including inebilizumab, satralizumab, eculizumab (12–16). However, the widespread clinical use of biologics has been limited by issues of accessibility and cost. Currently, traditional immunosuppressants (ISTs) such as oral mycophenolate mofetil (MMF) and azathioprine (AZA), as well as the intravenous anti-CD20 monoclonal antibody rituximab (RTX), are still used off-label to prevent NMOSD relapses in developing countries like China (17). The risk–benefit profile of those therapies remains under investigation. Therefore, evaluating the efficacy of ISTs and RTX among NMOSD populations, particularly in Chinese patients, is still necessary. Moreover, previous clinical studies have predominantly focused on clinical relapses and Expanded Disability Status Scale (EDSS) scores rather than magnetic resonance imaging (MRI) activity. In this study, we aimed to comprehensively evaluate the efficacy of AZA, MMF, TAC, and RTX in NMOSD by considering not only post-treatment clinical relapses as the primary outcome but also analyzing relapse-independent MRI activity.

## Methods

### Study design and population

This study retrospectively analyzed 113 NMOSD patients from the Department of Neurology at the First Affiliated Hospital of Chongqing Medical University and 100 NMOSD patients from the Department of Neurology at Beijing Tiantan Hospital between September 1, 2014, and August 31, 2023. At the time of database lock, the cohort comprised 213 NMOSD patients. Inclusion criteria were: (1) diagnosis according to the 2015 revised NMOSD criteria by Wingerchuk (18), and no exclusion of patients with comorbid autoimmune diseases; (2) treatment with oral conventional immunosuppressants (ISTs) such as AZA, MMF, TAC, or RTX; and (3) treatment duration of at least 6 months. Exclusion criteria were: (1) use of other immunotherapies, excluding oral corticosteroids, within 3 months prior to treatment initiation; and (2) incomplete clinical data or loss to follow-up.

### Data collection

Clinical and imaging data were collected from the inpatient Health Information System (HIS) at the study centers, supplemented by regular outpatient visits and telephone follow-up. Data collected included gender, age at onset, disease duration, AQP4-IgG status (a standardized cell-based assay using indirect immunofluorescence with anti-human IgG), initial symptoms, comorbid autoimmune diseases, and adverse reactions.

### Outcome measurement

The cohort was divided into two datasets: Dataset A, comprising 213 patients for clinical activity analysis, and Dataset B, including 81 patients for both clinical and MRI activity analysis. The primary efficacy outcome was time to the first relapse post-immunotherapy. Secondary outcomes included relapse rate, MRI activity, drug persistence, and annualized relapse rate (ARR). Safety was assessed by the occurrence of adverse events (AEs), defined as any event during drug treatment, and severe drug-related adverse events, necessitating hospitalization (2, 19).

### Clinical evaluation

In this study, relapse was defined as the appearance of new neurological deficit symptoms lasting more than 24 h or the worsening of pre-existing symptoms (14), occurring at least 30 days after the last acute attack and without other identifiable causes. Clinical activity was indicated by the occurrence of relapses. MRI activity was identified by the detection of at least one T1 gadolinium-enhancing lesion on brain or spinal cord MRI scans at least 12 months post-immunotherapy (20, 21). Relapse rate (RR) is defined as the proportion of patients experiencing their first disease recurrence within a specified period after initiating immunotherapy. Treatment failure was defined as the occurrence of at least one clinical relapse during the follow-up period, whereas treatment success was the absence of such relapses (22). Drug persistence was defined as the continuation of the prescribed treatment without discontinuation or switching during the follow-up period (23). The annualized relapse rate (ARR) was calculated by dividing the total number of relapses by the observed annual duration. To mitigate bias from elevated ARR due to early immunotherapy, patients with a disease duration ≤3 months were excluded from the pre-treatment ARR calculations (24). The disease course was defined as the duration from the first attack to 1 day before the initiation of long-term immunotherapy. Disability progression was defined as an increase of 1.5 points if the baseline EDSS score was 0; an increase of ≥1 point if the baseline EDSS score was between 1 and 5; and an increase of ≥0.5 points if the baseline EDSS score was ≥5.5 (25, 26).

### Ethical approval

The study received approval from the Ethics Committees of Beijing Tiantan Hospital and the First Affiliated Hospital of Chongqing Medical University (No. 2024ER789-1). Given the retrospective and non-interventional nature of the study, written informed consent was not required from patients. The reporting of this research was done in conjunction with the STROBE (Strengthening the Reporting of Observational Studies in Epidemiology) reporting guideline for cohort studies.

### Statistical analysis

SPSS 27.0 software (IBM, USA) was utilized for data analysis. The demographic and clinical characteristics of all patients were analyzed, and regression analysis was performed among clinical indicators. Measurement data were described using means (standard deviation), medians (range), and upper and lower quartiles, while categorical data were described using counts and percentages. Inter-group comparisons of measurement data utilized T-tests or nonparametric tests, while comparisons of categorical data employed chi-square tests. The Bonferroni method was used to adjust the significance level for these comparisons. The time to the first post-treatment relapse was analyzed using Kaplan–Meier (K-M) analysis, and inter-group comparisons utilized the Breslow method. In all cases, statistical significance was defined as a two-sided *p*-value <0.05. Partial statistical analyses used R software version 3.6.3, employing the “ggalluvial” and “ggplot2” packages for visualization, with statistical significance set at *p* < 0.05.

## Results

### Population

A total of 213 NMOSD patients were enrolled in the study, of whom 89.7% (191/213) were female. Among these patients, 82.6% (176/213) tested positive for AQP4-IgG, 12.2% (26/213) tested negative, and 5.2% (11/213) had indeterminate results. The median age of onset was 36 years (range: 13–80 years). IST mainly consisted of TAC, MMF, and AZA, accounting for 11.27% (24/213), 34.74% (74/213), and 15.96% (34/213), respectively, while RTX accounted for 38.03% (81/213). In the IST group, 90.2% (119/132) of patients received immunotherapy for the first time, whereas 9.8% (13/132) had received other immunotherapies within the 3 months prior to treatment. Among patients treated with RTX, 67.9% (55/81) had previously used other immunosuppressants. There were no significant differences between the two groups in terms of gender, age of onset, follow-up duration, comorbid autoimmune diseases, AQP4-IgG positivity rate, presenting symptoms, number of relapses in the year prior to treatment, pre-treatment ARR, and pre- and post-treatment EDSS scores (*p* > 0.05). Table 1 presents a detailed description of the demographic and baseline characteristics.

**Table 1:** Clinical characteristics of patients treated with rituximab (RTX), tacrolimus (TAC), mycophenolate mofetil (MMF), and azathioprine (AZA).

Characteristic	RTX (*n* = 81)	IST (*n* = 132)	†*p* value	IST			‡*p* value
				TAC (*n* = 24)	MMF (*n* = 74)	AZA (*n* = 34)	
Age at disease onset, years, median (IQR)	35.00 [22.00, 48.00]	37.00 [25.75, 49.00]	0.322	38.00 [25.75, 47.25]	36.00 [25.25, 49.00]	38.00 [28.00, 48.75]	0.772
Disease duration before treatment initiation, years, median (IQR)	3.00 [0.58, 5.50]	1.71 [0.08, 4.02]	0.028	2.00 [0.00, 4.17]	1.67 [0.19, 4.06]	1.54 [0.12, 4.00]	0.183
Duration of treatment, months, median (IQR)	27.00 [19.00, 46.00]	37.00 [24.00, 62.50]	0.001	29.50 [23.25, 37.00]	40.00 [30.25, 63.50]	39.00 [24.00, 69.00]	0.001
Follow-up time, weeks, median (IQR)	104.00 [70.00, 168.00]	104.00 [83.75, 198.00]	0.112	104.00 [46.25, 104.00]	106.00 [101.00, 246.00]	104.00 [72.50, 239.00]	0.007
ARR before treatment, mean (95% CI)	0.92 [0.69–1.15]	0.86 [0.66–1.06]	0.092	1.10 [0.54–1.66]	1.10 [0.77–1.42]	0.38 [0.23–0.53]	<0.001
ARR after treatment, mean (95% CI)	0.12 [0.03–0.21]	0.18 [0.12–0.23]	0.157	0.21 [0.03–0.39]	0.15 [0.07–0.23]	0.19 [0.08–0.3]	0.081
EDSS before treatment	4.00 [2.50, 6.00]	3.25 [2.00, 4.50]	0.079	3.00 [2.38, 4.00]	3.25 [2.00, 4.38]	4.00 [2.00, 5.00]	0.202
EDSS after treatment	3.00 [2.00, 4.50]	3.00 [2.00, 4.50]	0.859	3.00 [2.00, 4.00]	3.00 [2.00, 4.38]	4.00 [2.00, 4.50]	0.697
Time to first recurrence after treatment, days, median (IQR)	0.00 [0.00, 0.00]	0.00 [0.00, 270.00]	0.017	0.00 [0.00, 276.00]	0.00 [0.00, 0.00]	0.00 [0.00, 395.00]	0.039
Sex, n (%)			0.146				0.359
Female	12 (14.8)	10 (7.6)		1 (4.2)	6 (8.1)	3 (8.8)	
Male	69 (85.2)	122 (92.4)		23 (95.8)	68 (91.9)	31 (91.2)	
Concomitant autoimmune diseases, n (%)			1.000				0.650
No	72 (88.9)	118 (89.4)		23 (95.8)	66 (89.2)	29 (85.3)	
Yes	9 (11.1)	14 (10.6)		1 (4.2)	8 (10.8)	5 (14.7)	
AQP4 antibody, n (%)			0.050				0.347
Anti-AQP4 antibody negative	10 (12.3)	16 (12.1)		2 (8.3)	10 (13.5)	4 (11.8)	
Anti-AQP4 antibody positive	63 (77.8)	113 (85.6)		21 (87.5)	63 (85.1)	29 (85.3)	
Unknown	8 (9.9)	3 (2.3)		1 (4.2)	1 (1.4)	1 (2.9)	
Onset, n (%)			0.038				<0.001
Supratentorial	6 (7.4)	7 (5.3)		2 (8.3)	1 (1.4)	4 (11.8)	
Optic neuritis	4 (4.9)	2 (1.5)		0 (0.0)	0 (0.0)	2 (5.9)	
Brainstem	4 (4.9)	0 (0.0)		0 (0.0)	0 (0.0)	0 (0.0)	
Myelitis	19 (23.5)	48 (36.4)		1 (4.2)	33 (44.6)	14 (41.2)	
Mixed	30 (37.0)	45 (34.1)		9 (37.5)	25 (33.8)	11 (32.4)	
Absence	18 (22.2)	30 (22.7)		12 (50.0)	15 (20.3)	3 (8.8)	
Onset symptoms			0.059				0.147
Visual impairment	35 (43.2)	37 (28.0)		9 (37.5)	19 (25.7)	9 (26.5)	
Sensory disturbance	13 (16.0)	17 (12.9)		3 (12.5)	12 (16.2)	2 (5.9)	
Limb weakness	5 (6.2)	5 (3.8)		2 (8.3)	1 (1.4)	2 (5.9)	
Vomiting and hiccups	0 (0.0)	2 (1.5)		0 (0.0)	2 (2.7)	0 (0.0)	
Mixed	28 (34.6)	68 (51.5)		9 (37.5)	39 (52.7)	20 (58.8)	
Cranial nerve palsy	0 (0.0)	3 (2.3)		1 (4.2)	1 (1.4)	1 (2.9)	
Previous immunotherapy			<0.001				<0.001
No	26 (32.1)	119 (90.2)		21 (87.5)	64 (86.5)	34 (100.0)	
Yes	55 (67.9)	13 (9.8)		3 (12.5)	10 (13.5)	0 (0.0)	
Concurrent use of prednisolone, n (%)			<0.001				<0.001
No	70 (86.4)	41 (31.1)		21 (87.5)	19 (25.7)	1 (2.9)	
Yes	11 (13.6)	91 (68.9)		3 (12.5)	55 (74.3)	33 (97.1)	
Number of relapses 1 year before treatment, n (%)			0.465				0.607
0	33 (40.7)	61 (46.2)		11 (45.8)	32 (43.2)	18 (52.9)	
1	38 (46.9)	54 (40.9)		11 (45.8)	32 (43.2)	11 (32.4)	
2	8 (9.9)	12 (9.1)		2 (8.3)	8 (10.8)	2 (5.9)	
3	1 (1.2)	5 (3.8)		0 (0.0)	2 (2.7)	3 (8.8)	
4	1 (1.2)	0 (0.0)		0 (0.0)	0 (0.0)	0 (0.0)	
Discontinuation/Switch of prescription			<0.001				<0.001
Continuation of prescription	71 (87.7)	78 (59.1)		12 (50.0)	54 (73.0)	12 (35.3)	
Discontinuation of prescription	7 (8.6)	25 (18.9)		7 (29.2)	9 (12.2)	9 (26.5)	
Switch of prescription	3 (3.7)	29 (22.0)		5 (20.8)	11 (14.9)	13 (38.2)	
Treatment outcome			0.029				0.016
Success	67 (82.7)	90 (68.2)		14 (58.3)	56 (75.7)	20 (58.8)	
Failure	14 (17.3)	42 (31.8)		10 (41.7)	18 (24.3)	14 (41.2)	

Presents a detailed description of the demographic and baseline characteristics.

RTX, Rituximab; IST, Immunosuppressive Therapy (includes TAC, MMF, AZA); TAC, Tacrolimus; MMF, Mycophenolate Mofetil; AZA, Azathioprine; ARR, Annualized Relapse Rate; EDSS, Expanded Disability Status Scale; AQP4, aquaporin 4; HR, Hazard Ratio; CI, Confidence interval; IQR, Interquartile Range.

Statistical Analysis: Continuous variables: Median [interquartile range, IQR] or mean (95% CI); categorical variables: counts (%). † *p* value: Comparisons between RTX and IST groups. ‡ *p* value: Comparisons among the RTX, TAC, MMF, and AZA groups.

### Protocol of treatment

The prescription details are as follows: 24 patients received TAC treatment, among whom 87.5% (21/24) tested positive for AQP4-IgG. Studies have shown that tacrolimus blood concentration is influenced by certain foods and drugs (e.g., carbamazepine, grapefruit). Therefore, monitoring the tacrolimus concentration is crucial, as it helps physicians assess the drug’s efficacy and adjust the dosage. TAC dosage distribution included: 66.67% (16/24) received 2 mg/qd (once daily), 8.33% (2/24) received 1 mg/bid (twice daily), 12.5% (3/24) received 3 mg/qd, 4.17% (1/24) received 4 mg/qd, and 8.33% (2/24) received 0.5 mg/bid. A total of 74 patients received MMF treatment, of whom 85.1% (63/74) tested positive for AQP4-IgG. Of the MMF patients, 51.35% (38/74) were on 500 mg/bid, 37.84% (28/74) on 750 mg/bid, 4.05% (3/74) on 1,000 mg/qd, and 4.05% (3/74) on 250 mg/bid. A total of 34 patients received AZA treatment, of whom 85.3% (29/34) tested positive for AQP4-IgG. Among them, 58.82% (20/34) received 100 mg/qd, 23.53% (8/34) received 50 mg/bid, 5.89% (2/34) received 50 mg/qd, and 5.89% (2/34) received 25 mg/bid. Additionally, 97.1% (33/34) of patients in the AZA group routinely received low-dose corticosteroid therapy (5–10 mg/qd), 74.3% (55/74) of patients in the MMF group partially received low-dose corticosteroid therapy, while 12.5% (3/24) of patients in the TAC group did not routinely receive low-dose corticosteroid therapy. A total of 81 patients received RTX treatment, of whom 77.8% (63/81) tested positive for AQP4-IgG. The RTX administration regimen consisted of intravenous infusions: 100 mg on the first day and 500 mg on the second day. After a two-week interval, a loading dose of 500 mg was given. Subsequent dosing intervals were adjusted based on peripheral blood B-cell levels during follow-up, with 500 mg administered every 6 months. Additionally, 13.6% (11/81) of patients in the RTX group received low-dose corticosteroid maintenance therapy. Compared to the RTX group, combined corticosteroid therapy was more common in the IST group (68.9%, 91/132).

### Efficacy

**Figure 1:**
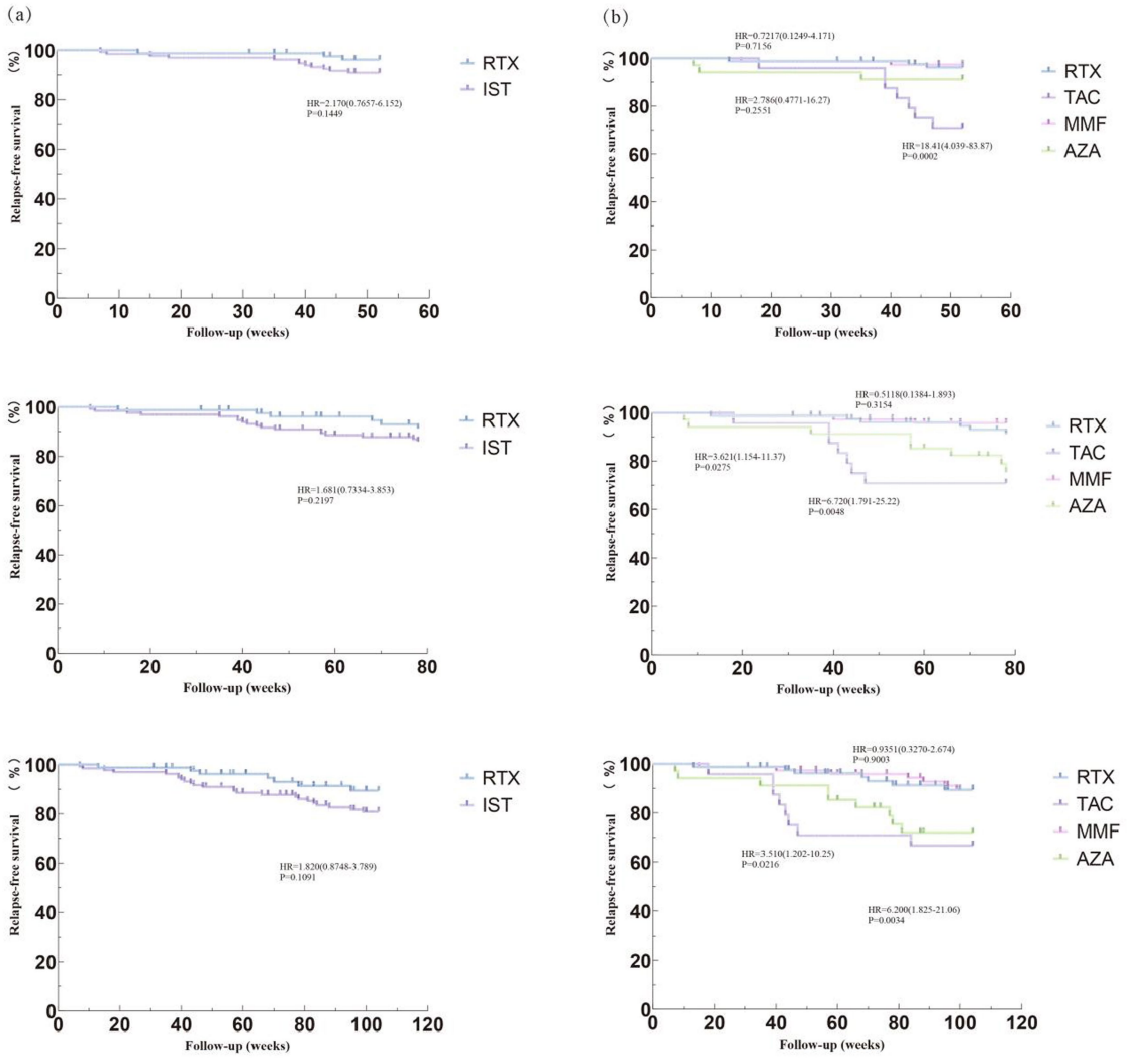
Compare relapse – free survival rates among different treatment groups. Image shows the Kaplan–Meier (K-M) method describing the relapse-free survival rates. **(a)** Comparing the risk of first recurrence after 1 year, 1.5 years, and 2 years of immunotherapy between the RTX group and the IST group. **(b)** Comparing the risk of first recurrence after 1 year, 1.5 years, and 2 years of immunotherapy among the RTX group, TAC group, AZA group, and MMF group. RTX, Rituximab; IST, Immunosuppressive Therapy (includes TAC, MMF, AZA); TAC, Tacrolimus; MMF, Mycophenolate Mofetil; AZA, Azathioprine; HR, Hazard Ratio; CI, Confidence interval; IQR, Interquartile Range.

In Dataset A, a comparison of 213 NMOSD patients was performed to evaluate drug efficacy. The time to first relapse after treatment was used as the primary outcome for each group. The results showed that after 1 year of immunotherapy, the risk of first relapse was significantly higher in the TAC group compared to the RTX and MMF groups, with hazard ratios (HRs) of 18.41 (95% CI: 4.039–83.87; *p* < 0.05) and 22.72 (95% CI: 4.783–108.0; *p* < 0.0001), respectively. The risk of first relapse in the AZA group was higher compared to the RTX group (HR = 2.786, 95% CI: 0.4771–16.27; *p* = 0.2551) and the MMF group (HR = 4.005, 95% CI: 0.5973–26.86; *p* = 0.1529), although the differences were not statistically significant. After 2 years of immunotherapy, the risk of first relapse in the TAC group was significantly higher than that in the RTX group (HR = 6.200, 95% CI: 1.825–21.06; *p* = 0.0034) and the MMF group (HR = 6.017, 95% CI: 1.782–20.32; *p* = 0.0039). Compared to the RTX group, the risk of first relapse after treatment was significantly higher in the AZA group, with an HR of 3.510 (95% CI: 1.202–10.25; *p* = 0.0216). Similarly, the risk was significantly higher in the AZA group compared to the MMF group, with an HR of 3.909 (95% CI: 1.318–11.59; *p* = 0.014). Additionally, compared to the RTX group, the overall IST group exhibited a higher risk of first relapse; however, this difference did not reach statistical significance. The HR at 1 year was 2.170 (95% CI: 0.7657–6.152; *p* = 0.1449), and at 2 years, it was 1.82 (95% CI: 0.8748–3.789; *p* = 0.1091). Figure 1 shows the Kaplan–Meier method describing the time to first recurrence after treatment.

The relapse rates after immunotherapy were analyzed. The median follow-up duration was 104 weeks (range: 12–336) for the RTX group and 104 weeks (range: 8–596) for the IST group. In the RTX group, 82.7% (67/81) of patients achieved treatment success, while 17.3% (14/81) experienced treatment failure. In the IST group, 68.2% (90/132) of patients achieved treatment success, and 31.8% (42/132) experienced treatment failure. A significant difference in clinical relapses was observed between the IST and RTX groups (*p* = 0.029). Additionally, the relapse rate in the RTX group was lower than that in the MMF group, with a significant difference between the two groups (*p* = 0.032). Significant differences were also noted between the RTX group and the TAC and AZA groups (*p* < 0.001).

In Dataset A, the annualized relapse rate (ARR) in the IST group before treatment (excluding patients treated within 3 months after the first attack) was 0.86 (95% CI: 0.66–1.06), significantly higher than the post-treatment ARR of 0.18 (95% CI: 0.12–0.23). Similarly, the ARR in the RTX group was 0.92 (95% CI: 0.69–1.15) before treatment, significantly higher than the post-treatment ARR of 0.12 (95% CI: 0.03–0.21). However, no significant difference in post-treatment ARR was observed between the RTX and IST groups (*p* = 0.157). All four drugs effectively reduced the ARR. RTX showed better disease relapse control (post-treatment ARR = 0.12, 95% CI: 0.03–0.21) compared to TAC (post-treatment ARR = 0.21, 95% CI: 0.03–0.39), MMF (post-treatment ARR = 0.15, 95% CI: 0.07–0.23), and AZA (post-treatment ARR = 0.19, 95% CI: 0.08–0.30), although no significant differences were found between the groups (*p* = 0.081).

An analysis of pre- and post-treatment EDSS scores in the IST and RTX groups showed improvement in both groups. However, no statistically significant differences were found between the groups (*p* > 0.05). Among the 157 patients with no relapses, 6.37% (10/157) experienced disability progression, while 93.63% (147/157) maintained stable or improved EDSS scores. Univariate analysis showed that, compared to patients treated with RTX, those treated with TAC had a significantly lower proportion of patients without relapses (HR = 2.57, 95% CI: 1.10–5.99; *p* = 0.029). In multivariate analysis, after adjusting for factors such as age at onset, sex, AQP4 antibody status, site of onset, pre-treatment ARR, pre-treatment disease duration, corticosteroid maintenance therapy, comorbid autoimmune diseases, and pre-treatment EDSS score, no significant differences were observed between the RTX group and the other groups. Both univariate and multivariate analyses showed that patients with myelitis had a lower risk of relapse compared to those with supratentorial involvement. No significant differences were observed for other factors, likely due to the limited sample size in this study. Tables 2, 3 provide detailed descriptions of the univariate and multivariate analyses, respectively.

**Table 2:** Factors associated with recurrence risk after initiating RTX and IST therapies.

Factor	Univariate analysis		Multivariate analysis	
	HR (95% CI)	*p*	HR (95% CI)	*p*
First-line treatment				
RTX, *n* = 81	Reference		Reference	
IST, *n* = 132	1.43 (0.78, 2.63)	0.253	1.25 (0.61, 2.58)	0.542
Sex				
Female, *n* = 22	Reference		Reference	
Male, *n* = 191	0.99 (0.42, 2.31)	0.976	1.30 (0.52, 3.28)	0.577
Age at disease onset, years	0.99 (0.97, 1.01)	0.243	0.99 (0.97, 1.01)	0.538
AQP4 antibody				
Anti-AQP4 antibody negative	Reference		Reference	
Anti-AQP4 antibody positive	0.61 (0.31, 1.21)	0.159	0.79 (0.37, 1.66)	0.529
Onset				
Supratentorial	Reference		Reference	
Optic neuritis	0.32 (0.04, 2.62)	0.289	0.41 (0.05, 3.73)	0.429
Brainstem	0.30 (0.04, 2.40)	0.254	0.36 (0.04, 3.09)	0.349
Myelitis	0.27 (0.11, 0.65)	0.004	0.28 (0.10, 0.73)	0.009
Mixed	0.61 (0.26, 1.38)	0.234	0.60 (0.25, 1.43)	0.249
ARR before treatment	0.94 (0.71, 1.25)	0.688	1.00 (0.74, 1.36)	0.985
Disease duration before treatment initiation, years	1.02 (0.97, 1.07)	0.479	1.01 (0.95, 1.06)	0.805
Concurrent use of prednisolone				
No	Reference		Reference	
Yes	1.01 (0.59, 1.74)	0.968	1.17 (0.61, 2.25)	0.637
Concomitant autoimmune diseases				
No	Reference		Reference	
Yes	0.52 (0.16, 1.67)	0.271	0.61 (0.18, 2.07)	0.424
EDSS before treatment	0.95 (0.83, 1.08)	0.426	0.95 (0.82, 1.10)	0.464

RTX, Rituximab; IST, Immunosuppressive Therapy (includes TAC, MMF, AZA); ARR, Annualized Relapse Rate; EDSS, Expanded Disability Status Scale; AQP4, aquaporin 4; HR, Hazard Ratio; CI, Confidence interval. Analysis Purpose: Univariate Analysis: To assess the individual association of each variable (e.g., treatment type, age, sex) with relapse risk, without adjusting for confounding factors. Multivariate Analysis: To evaluate the independent effect of variables on relapse risk after adjusting for covariates (age, sex, AQP4 antibody status, baseline EDSS, and disease duration). Model: Cox proportional hazards regression. Significance Threshold: *p* < 0.05.

**Table 3:** Factors associated with recurrence risk following the initiation of RTX, TAC, MMF, and AZA therapies.

Factor	Univariate analysis		Multivariate analysis	
	HR (95% CI)	*p*	HR (95% CI)	*p*
First-line treatment				
RTX, *n* = 81	Reference		Reference	
TAC, *n* = 24	2.57 (1.10, 5.99)	0.029	2.00 (0.81, 4.98)	0.135
MMF, *n* = 74	1.01 (0.50, 2.03)	0.981	0.85 (0.36, 2.00)	0.710
AZA, *n* = 34	1.90 (0.89, 4.05)	0.096	1.54 (0.55, 4.37)	0.414
Sex				
Female, *n* = 22	Reference		Reference	
Male, *n* = 191	0.99 (0.42, 2.31)	0.976	1.22 (0.49, 3.05)	0.670
Age at disease onset, years	0.99 (0.97, 1.01)	0.243	0.99 (0.97, 1.01)	0.538
AQP4 antibody				
Anti-AQP4 antibody negative	Reference		Reference	
Anti-AQP4 antibody positive	0.61 (0.31, 1.21)	0.159	0.74 (0.35, 1.56)	0.422
Onset				
Supratentorial	Reference		Reference	
Optic neuritis	0.32 (0.04, 2.62)	0.289	0.42 (0.04, 3.90)	0.444
Brainstem	0.30 (0.04, 2.40)	0.254	0.44 (0.05, 3.87)	0.457
Myelitis	0.27 (0.11, 0.65)	0.004	0.33 (0.13, 0.88)	0.027
Mixed	0.61 (0.26, 1.38)	0.234	0.69 (0.29, 1.65)	0.404
ARR before treatment	0.94 (0.71, 1.25)	0.688	1.05 (0.77, 1.42)	0.763
Disease duration before treatment initiation, years	1.02 (0.97, 1.07)	0.479	1.01 (0.96, 1.07)	0.712
Concurrent use of prednisolone				
No	Reference		Reference	
Yes	1.01 (0.59, 1.74)	0.968	1.32 (0.57, 3.05)	0.513
Concomitant autoimmune diseases				
No	Reference		Reference	
Yes	0.52 (0.16, 1.67)	0.271	0.62 (0.18, 2.14)	0.445
EDSS before treatment	0.95 (0.83, 1.08)	0.426	0.94 (0.81, 1.09)	0.417

RTX, Rituximab; TAC, Tacrolimus; MMF, Mycophenolate Mofetil; AZA, Azathioprine; ARR, Annualized Relapse Rate; EDSS, Expanded Disability Status Scale; AQP4, aquaporin 4; HR, Hazard Ratio; CI, Confidence interval. Analysis Purpose: Univariate Analysis: To assess the individual association of each variable (e.g., treatment type, age, sex) with relapse risk, without adjusting for confounding factors. Multivariate Analysis: To evaluate the independent effect of variables on relapse risk after adjusting for covariates (age, sex, AQP4 antibody status, baseline EDSS, and disease duration). Model: Cox proportional hazards regression. Significance Threshold: *p* < 0.05.

**Figure 2:**
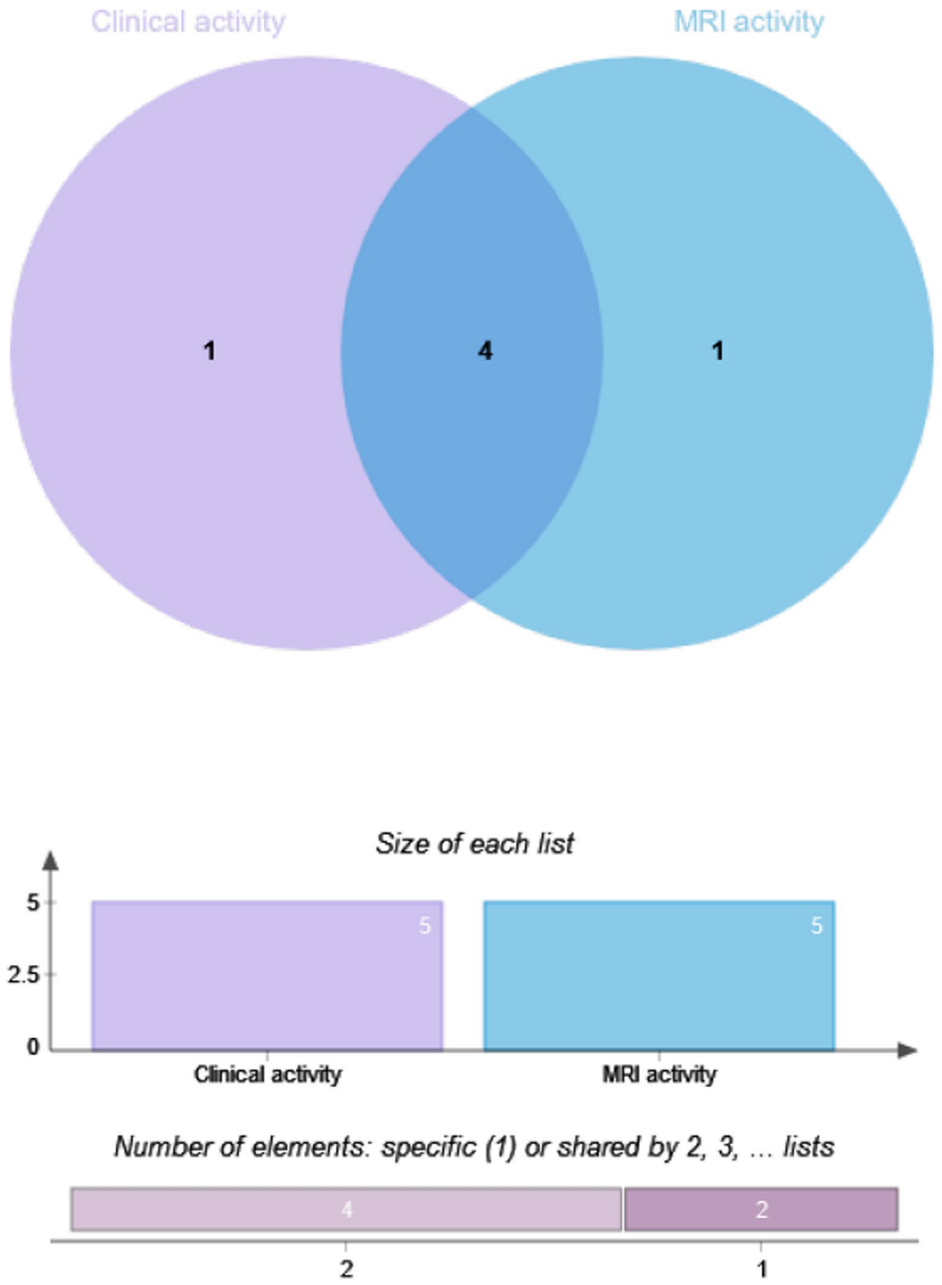
Overlap analysis of clinical and MRI activity in NMOSD patients by RTX group.

**Figure 3:**
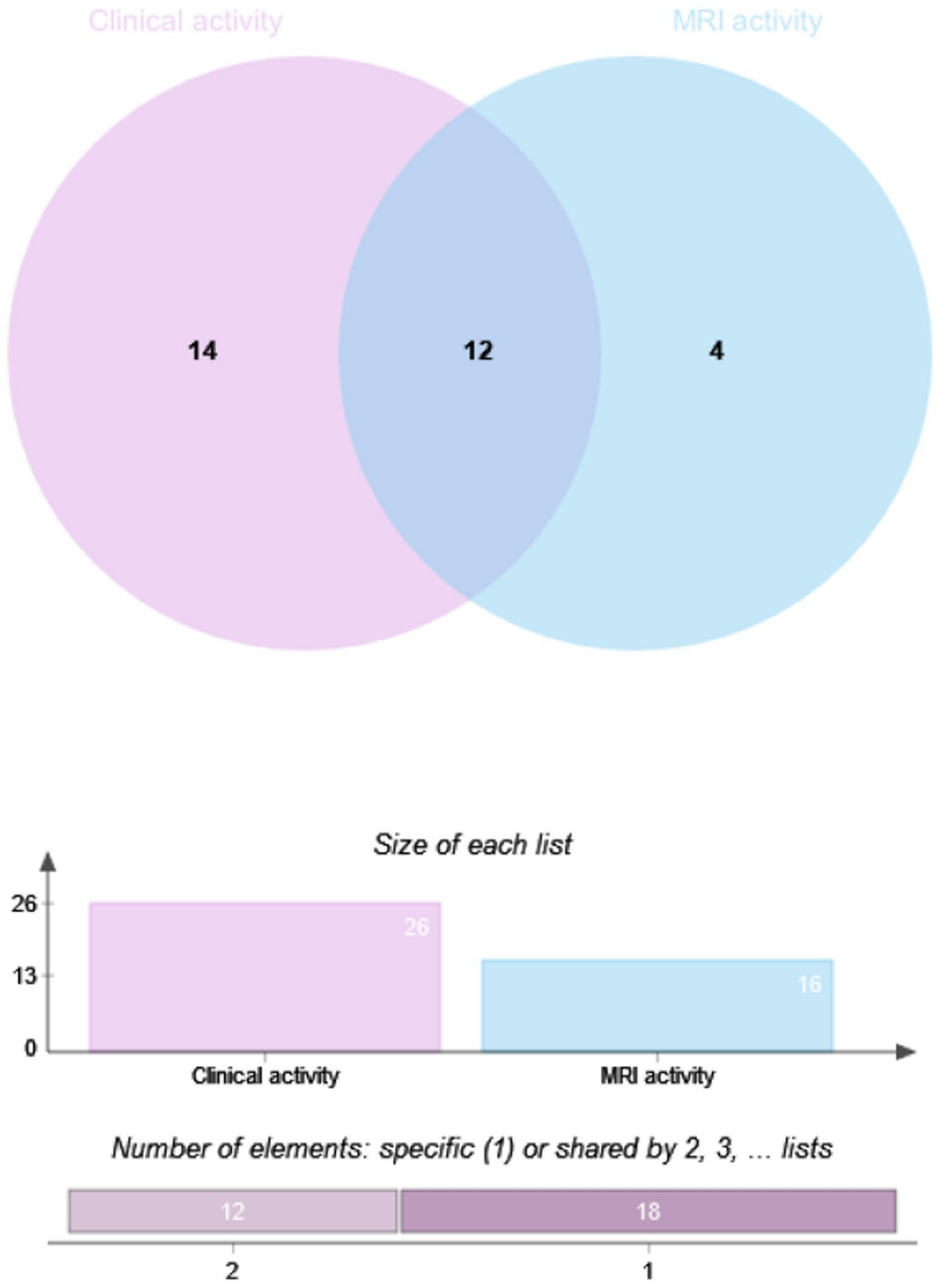
Overlap analysis of clinical and MRI activity in NMOSD patients by IST group. RTX, Rituximab; IST, Immunosuppressive Therapy (includes TAC, MMF, AZA); MRI, Magnetic Resonance Imaging. Method: Overlap analysis visualized via Venn diagrams; intergroup differences assessed using Fisher’s exact test. Significance Threshold: *p* < 0.05.

The drug efficacy in 81 patients from Dataset B was compared. These patients underwent MRI scans at least 12 months post-treatment, with 28.4% (23/81) in the RTX group and 71.6% (58/81) in the IST group. When MRI activity alone was used as the outcome for Dataset B, 21.74% (5/23) of patients in the RTX group had T1-weighted imaging (T1WI)-enhancing lesions, compared to 27.59% (16/58) in the IST group. When clinical activity alone was assessed, 44.8% (26/58) of patients in the IST group experienced relapses, compared to 21.7% (5/23) in the RTX group. When clinical and MRI activities were combined, 51.7% (30/58) of patients in the IST group had disease activity, compared to 26.1% (6/23) in the RTX group. A statistically significant difference in post-treatment disease activity was observed between the RTX and IST groups, with disease activity being significantly lower in the RTX group (*p* = 0.036). Table 4 and Figures 2, 3 provide detailed information on the disease activity of these 81 patients during the follow-up period.

**Table 4:** Disease activity during the follow-up period was assessed by different methods.

Characteristic	Whole population (*n* = 81)	IST, *n* = 58	RTX, *n* = 23	*p*
Clinical relapse, n (%)	31 (38.3)	26 (44.8)	5 (21.7)	0.054
MRI activity, n (%)	21 (25.9)	16 (27.6)	5 (21.7)	0.588
Relapse (clinical and MRI), n (%)	36 (44.4)	30 (51.7)	6 (26.1)	0.036

RTX, Rituximab; IST, Immunosuppressive Therapy (includes TAC, MMF, AZA); MRI, Magnetic Resonance Imaging. Analysis Purpose: Compare disease activity (clinical relapse, MRI activity, and combined relapse) between IST and RTX groups. Threshold: *p* < 0.05.

### Persistence and switched treatments

Drug persistence was analyzed for all patients in the dataset. The results showed that 15.0% (32/213) of patients discontinued their medication during the follow-up period, while 15.0% (32/213) switched to another prescription. Specifically, 8.6% (7/81) of patients in the RTX group discontinued their medication. In the TAC group, 29.2% (7/24) discontinued, compared to 12.2% (9/74) in the MMF group and 26.5% (9/34) in the AZA group. In the RTX group, 3.7% (3/81) switched to another prescription, compared to 20.8% (5/24) in the TAC group, 14.9% (11/74) in the MMF group, and 38.2% (13/34) in the AZA group. In the IST group, 40.9% (54/132) of patients discontinued their medication, compared to only 12.3% (10/81) in the RTX group. The proportion of RTX patients who discontinued or switched prescriptions was significantly lower than that of the IST group (*p* < 0.001).

**Figure 4:**
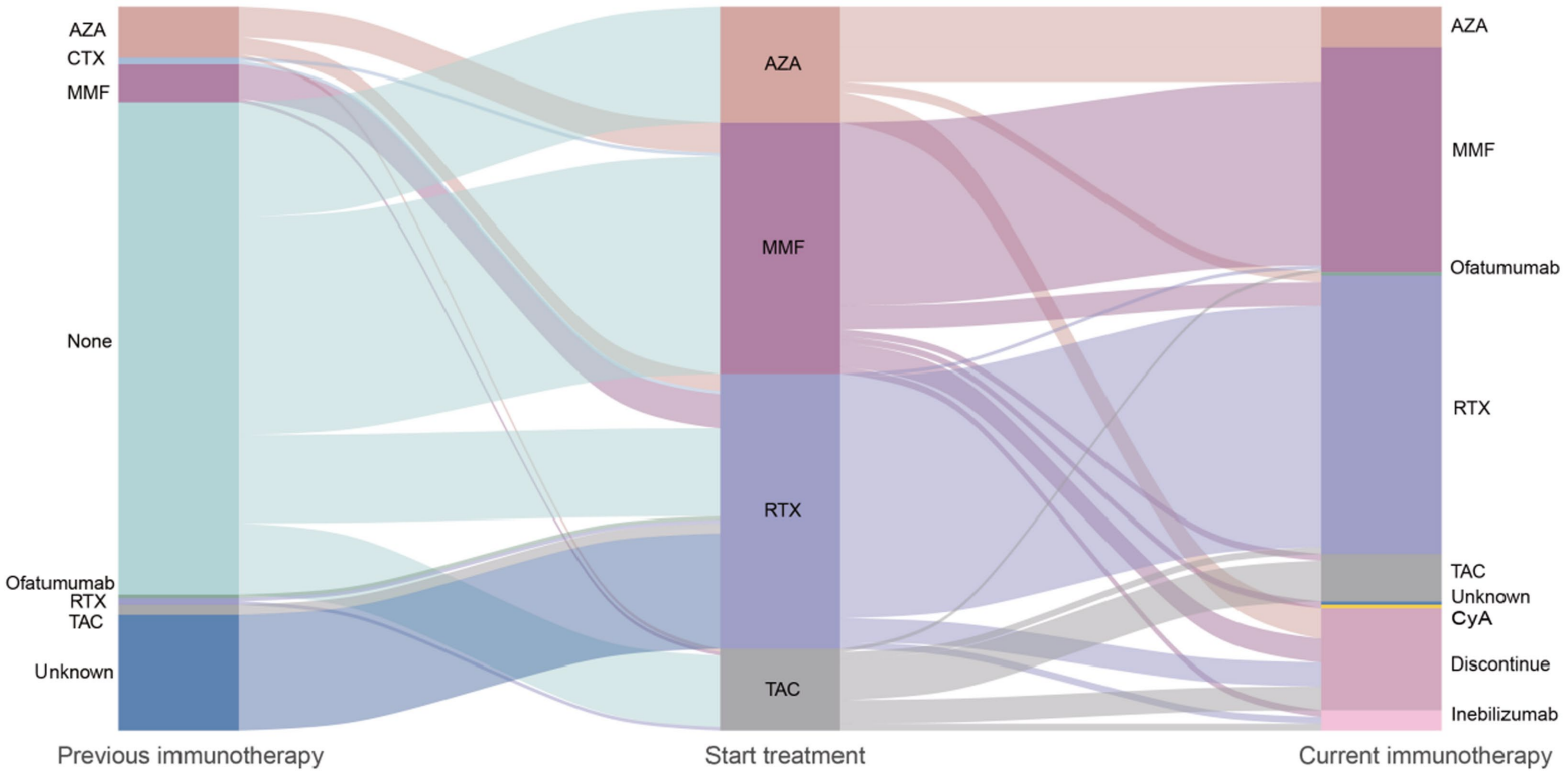
Flowchart of immunotherapy transitions in NMOSD patients: treatment initiation, maintenance, and discontinuation. AZA, Azathioprine; CTX, Cyclophosphamide; MMF, Mycophenolate Mofetil; RTX, Rituximab; TAC, Tacrolimus; Inebilizumab, Anti-CD19 monoclonal antibody; CyA, Cyclosporine A; ofatumumab, Anti-CD20 monoclonal antibody. Immunotherapies applied in the 213 patients with aquaporin 4 antibody-positive neuromyelitis optica spectrum disorder and treatment changes over time. The width of each flow represents proportionally to the flow quantity. Reflects the proportion of patients receiving each therapy. Patients without subsequent immunosuppressants remained on the previous treatment during the next transition. Method: Sankey diagram generated using R (ggalluvial package) to visualize treatment pathways.

Regarding the reasons for discontinuation and switching medications, the most common reason in the RTX group was personal preference to switch to switch drugs (60%, 6/10). In the IST group, 74% (40/54) of patients discontinued or switched medications due to post-treatment relapses or perceived lack of efficacy. Subgroup analysis of immunotherapy in the RTX group revealed significant differences in drug persistence between the RTX group and the TAC, MMF, and AZA groups (*p* < 0.05). Among ISTs, the AZA group had the lowest drug retention rate (35.3%, 12/34), with a significant difference observed between the MMF group (73%, 54/74) and the AZA group (*p* < 0.001). However, no statistically significant differences in drug persistence were observed between the TAC group (50%, 12/24) and the MMF or AZA groups. Table 5 and Figure 4 provide details on medication discontinuation and switching among patients.

**Table 5:** Reasons for immunotherapy discontinuation or prescription change in NMOSD patients by treatment group.

Discontinue/change prescription	RTX = 81	TAC = 24	MMF = 74	AZA = 34	*p*
Change prescription	3 (3.7)	5 (20.8)	11 (14.9)	13 (38.2)	*p* < 0.001
Poor efficacy	0 (0.0)	4 (16.7)	8 (10.8)	12 (35.5)	*p* < 0.001
Personal wishes	2 (2.5)	0 (0.0)	1 (1.4)	0 (0.0)	0.685
Pregnant	0 (0.0)	0 (0.0)	1 (1.4)	0 (0.0)	0.596
Other	1 (1.2)	1 (4.2)	1 (1.4)	1 (2.9)	0.757
Discontinue prescription	7 (8.6)	7 (29.2)	9 (12.2)	9 (26.5)	0.016
Poor efficacy	1 (1.2)	4 (16.7)	4 (5.4)	8 (23.5)	*p* < 0.001
Personal wishes	4 (4.9)	2 (8.3)	1 (1.4)	1 (2.9)	0.399
Pregnant	1 (1.2)	1 (4.2)	2 (2.7)	0 (0.0)	0.618
Other	1 (1.2)	0 (0.0)	2 (2.7)	0 (0.0)	0.628

RTX, Rituximab; TAC, Tacrolimus; MMF, Mycophenolate Mofetil; AZA, Azathioprine. Purpose: Compare reasons for treatment discontinuation or prescription changes across immunotherapy groups. Significance Threshold: *p* < 0.05.

### Safety

A safety evaluation was conducted for 213 patients during the follow-up period, with safety outcomes defined as hospitalization events related to adverse effects of immunotherapy. The results indicated that the safety profiles of RTX, TAC, MMF, and AZA were all tolerable, with no hospitalization events reported. The incidence of adverse reactions was 23.5% (8/34) in the AZA group, 16.2% (12/74) in the MMF group, 20.8% (5/24) in the TAC group, and 13.6% (11/81) in the RTX group. Abnormal liver function was the most common adverse reaction, but it resolved with liver-protective treatment. Table 6 provides a detailed description of specific adverse reactions observed during immunotherapy.

**Table 6:** A detailed description of specific adverse reactions observed during immunotherapy.

Adverse events	RTX, *n* = 81	TAC, *n* = 24	MMF, *n* = 74	AZA, *n* = 34	*p*
Incidence of adverse events, n (%)	11 (13.6)	5 (20.8)	12 (16.2)	8 (23.5)	0.575
Leukopenia, n (%)	0	1 (4.2)	0	1 (2.9)	0.131
Palpitations, n (%)	0	0	0	0	
Elevated transaminase levels, n (%)	4 (4.9)	1 (4.2)	1 (1.4)	3 (8.8)	0.335
Alopecia, n (%)	0	0	3 (4.1)	2 (5.9)	0.150
Infection, n (%)	4 (4.9)	0	0	0	0.084
Severe infection, n (%)	1 (1.2)	0	0	0	0.651
Rash, n (%)	0	1 (4.2)	1 (1.4)	0	0.271
Gastrointestinal disturbances, n (%)	0	0	4 (5.4)	0	0.054
Anemia, n (%)	2 (2.5)	0	0	0	0.349
Thrombocytopenia, n (%)	0	1 (4.2)	0	2 (5.9)	0.038
Onychomycosis, n (%)	0	0	0	0	
Odontoseisis, n (%)	0	0	0	0	
Tremor, n (%)	0	0	0	0	
Blood glucose elevate, n (%)	0	0	0	0	
Dyslipidemia, n (%)	0	0	0	0	
Weight gain, n (%)	0	0	1 (1.4)	0	0.596
Tumor, n (%)	0	1 (4.2)	2 (2.7)	0	0.278

RTX, Rituximab; TAC, Tacrolimus; MMF, Mycophenolate Mofetil; AZA, Azathioprine. Purpose: the incidence of adverse events was compared across four immunosuppressive therapy groups (RTX, TAC, MMF, AZA) in NMOSD patients. Threshold: *p* < 0.05.

## Discussion

This study compared multiple outcomes in NMOSD patients treated with TAC, MMF, AZA, and RTX, conducting a comprehensive evaluation of drug efficacy through clinical and MRI activity. Consistent with previous studies, both IST and RTX were shown to reduce NMOSD relapses (27–30). However, there is still controversy regarding the ranking of efficacy among different immunotherapies, which may be attributed to the heterogeneity of study designs, including differences in demographics, environmental factors, inclusion criteria, drug dosages, and frequencies. The main findings of this study are as follows: (1) RTX may be more effective than IST in treating NMOSD; (2) RTX and MMF may have better long-term efficacy than TAC and AZA; and (3) both RTX and IST are well-tolerated in NMOSD treatment.

The primary treatment goal is to prevent neurological deterioration, which is defined as no increase in EDSS scores. In our study, both time to first relapse and relapse rate after immunotherapy were used as outcomes. Survival analysis using time to first relapse showed that the risk of first relapse in the RTX group was lower than in the IST group during the first and second years of immunotherapy; however, this difference was not statistically significant. Subgroup analysis revealed that, in the first year after immunotherapy, the risk of first relapse in the TAC group was significantly higher than in the RTX and MMF groups (*p* < 0.05). In the second year, the risk of first relapse in the RTX and MMF groups was lower than in the TAC and AZA groups (*p* < 0.05). This suggests that TAC and AZA may be less effective than RTX and MMF.

When relapse after immunotherapy was used as an outcome, the relapse rate in the RTX group was significantly lower than in the IST group during the follow-up period. Specifically, the relapse rates in the TAC and AZA groups were higher, with a significant difference compared to RTX. However, no statistically significant differences in post-treatment relapse rates were found among the TAC, MMF, and AZA groups, although the relapse rate in the MMF group was lower than in the TAC and AZA groups. Consistent with the findings of Kim KH et al., our study demonstrated that RTX was superior to IST, and MMF may have better efficacy than AZA and TAC (31). The efficacy between AZA and TAC remains uncertain. Additionally, both the IST and RTX groups showed a significant reduction in ARR compared to pre-treatment levels. When calculating pre-treatment ARR, patients who received immunotherapy within 3 months of the initial onset were excluded, which resulted in smaller patient numbers in each IST subgroup.

Furthermore, no significant differences in EDSS scores before and after treatment were observed in NMOSD patients. Among the 157 patients who did not experience relapses, 10 cases of disability progression were identified through EDSS assessments. This phenomenon may be related to the pathogenic mechanisms of NMOSD, as subclinical disability progression is uncommon in NMOSD.

Imaging biomarkers have predictive value for relapse and prognosis in NMOSD. Studies have shown that brain MRI enhancement and spinal cord lesion length are associated with relapse risk in NMOSD patients (32–34). However, such studies are limited, and imaging biomarkers reflecting drug response are still lacking. Our study not only conducted comparative analyses using clinical relapse as an outcome but also analyzed post-treatment MRI activity. The results showed that when clinical activity was combined with brain and spinal cord MRI-enhancing lesions, the disease activity rate in both the RTX and IST groups was higher than when clinical activity alone was used as the outcome. However, compared to the IST group, the RTX group demonstrated significantly better control of MRI activity. This is consistent with Moog et al., who reported that high-efficacy treatments (biologics, primarily RTX) significantly reduced MRI progression in NMOSD patients compared to IST (35).

Furthermore, among patients with MRI enhancement but no clinical relapse after immunotherapy, the enhancement sites were at the same lesion sites as prior clinical events. Some studies have suggested that a reduction in MRI enhancement is closely associated with the efficacy of immunotherapy in multiple sclerosis patients (36). If MRI enhancement persists after immunotherapy, even without clinical relapse, it may indicate a higher relapse risk, suggesting suboptimal drug efficacy. Similarly, we hypothesize that the persistence of MRI enhancement without clinical relapse in NMOSD patients after immunotherapy may indicate suboptimal drug efficacy and a higher risk of relapse. Therefore, monitoring changes in MRI enhancement is crucial for evaluating disease activity and drug efficacy in NMOSD patients.

Although our results suggest that post-immunotherapy MRI activity cannot yet be used as an outcome to predict future relapses or long-term disability in NMOSD, this may be related to the short follow-up duration in our study or the characteristic lack of significant signal changes in MRI lesions over time in most NMOSD patients. However, long-term follow-up of NMOSD patients with post-treatment MRI-enhancing lesions is recommended to further investigate imaging biomarkers in NMOSD. This may help determine whether early adjustments to treatment regimens are necessary, particularly for patients without clinical relapse.

A statistical analysis of drug continuation rates between groups showed that the continuation rate in the IST group was significantly lower than in the RTX group (*p* < 0.001). In the IST group, 59.1% (78/132) of patients continued treatment, compared to 87.7% (71/81) in the RTX group. This discrepancy may be explained by the distinct administration schedules: the reduced frequency of RTX dosing likely diminishes treatment burden and enhances adherence. Moreover, the structured, clinician-monitored RTX regimen may foster patient engagement compared to self-managed daily pills, which are susceptible to non-adherence. While RTX is associated with infusion-related reactions primarily during initial doses, these events are typically transient and manageable. In contrast, daily oral ISTs may lead to cumulative gastrointestinal or hematologic side effects, contributing to higher discontinuation rates (37). Although long-term maintenance treatment plans for NMOSD patients should be based on personal preferences, 9.26% (5/54) of patients in the IST group and 60% (6/10) in the RTX group discontinued treatment due to personal preferences. In the RTX group, the primary reason for treatment discontinuation was stable disease status, leading to self-discontinuation, while the secondary reason was switching to newer drugs, such as inebilizumab.

We believe that drug persistence indirectly reflects treatment effectiveness. The higher continuation rate in the RTX group suggests that RTX may be more effective than IST in treating NMOSD. The AZA group had the highest discontinuation/switching rate, which may be related to the suboptimal efficacy of AZA. Additionally, AZA-related side effects may have contributed to treatment discontinuation. However, in this study, patients in the AZA group routinely received low-dose corticosteroid (5–10 mg/qd) maintenance therapy. Therefore, it is difficult to determine whether the high discontinuation rate in the AZA group was related to side effects from long-term oral corticosteroid use. A key limitation of this study is the lack of granular data on corticosteroid-specific toxicities. Although AZA patients received chronic low-dose corticosteroids, we could not systematically track complications such as bone mineral density loss or glycemic changes. Future studies should prospectively quantify corticosteroid-related adverse events to disentangle their contribution to treatment discontinuation. Additionally, comparing patients on IST with versus without corticosteroid co-therapy could clarify the independent effects of each agent. In contrast, RTX’s targeted mechanism reduces relapse rates with minimal corticosteroid dependence, thereby lowering cumulative toxicity. This aligns with studies showing that B lymphocyte (B cell) depletion therapies improve safety profiles by reducing steroid requirements in NMOSD (38).

Consistent with previous studies, RTX, TAC, MMF, and AZA all demonstrated good tolerability in our study. Due to the small number of patients in each drug subgroup, no patients were hospitalized or discontinued treatment due to adverse drug reactions. Additionally, the incidence of adverse reactions was significantly lower in the RTX group compared to the IST group. Among the IST subgroups, the incidence of adverse reactions was higher in the TAC and AZA groups than in the MMF group. The overall incidence of adverse reactions in each group was lower than that reported in previous studies (39, 40). We speculate that this may be related to the minimum treatment duration of >6 months for patients in our study, as most adverse drug reactions occur within 1–3 months of treatment initiation. If adverse drug reactions are not promptly managed, clinicians may switch medications during this period, leading to the exclusion of these patients from further analysis in our study. However, this design was intentional to ensure a homogeneous cohort for evaluating the long-term efficacy of RTX and IST. By excluding patients who could not tolerate the initial phase of treatment, we aimed to minimize confounding factors related to early treatment discontinuation and focus on the sustained therapeutic effects in patients who maintained the regimen beyond 6 months. To address this limitation, future studies should consider including all patients who received at least one dose (intent-to-treat analysis) and perform sensitivity analyses comparing completers versus early discontinuers. Additionally, our results are consistent with real-world clinical practice, where clinicians often discontinue or switch therapies in response to early adverse events.

Currently, FDA-approved first-line treatments for NMOSD, such as satralizumab, inebilizumab, and eculizumab, remain expensive. Compared to traditional immunosuppressants, novel monoclonal antibodies (mAbs) offer advantages in terms of efficacy and safety. However, their relatively high cost may limit widespread use in some regions. In China, many patients are not covered by insurance for these treatments, leading them to opt for more affordable off-label drugs like AZA, MMF, TAC, and RTX. Although RTX may be more effective than MMF in certain outcomes in our study, the differences did not reach statistical significance, and no definitive conclusions can be drawn. Future studies should further explore comparisons between novel monoclonal antibodies and traditional immunosuppressants, as well as their efficacy and safety in different NMOSD subtypes and disease stages.

The main limitations of this study include its non-blinded, retrospective design. The inconsistency in the duration and dosage of low-dose corticosteroids may hinder an accurate assessment of whether combined corticosteroid maintenance therapy affects the efficacy of RTX and ISTs. In our study, some patients receiving IST had previously been treated with other immunosuppressive drugs. Although we excluded patients who had received other immunotherapies within 3 months prior to treatment, the potential effects of prior immunotherapies cannot be completely ruled out. Additionally, due to the small sample size, we did not stratify the severity of relapses, making it impossible to compare the risk of severe relapses between different treatment groups. Furthermore, the inconsistency in MRI scanning equipment and parameters before and after treatment among NMOSD patients prevented direct comparison of MRI lesions. Finally, since the clinical application of RTX began later than IST, newly diagnosed patients were more likely to choose RTX as a treatment option, resulting in shorter follow-up durations in the RTX group compared to the IST group. Therefore, the long-term efficacy of different drugs could not be accurately evaluated. Despite these limitations, our study provides valuable treatment insights for NMOSD patients who cannot access newly approved biologics, such as inebilizumab and satralizumab.

In conclusion, our study contributes to the data on drug efficacy comparisons in the Chinese NMOSD cohort. We comprehensively evaluated and compared the effects of different ISTs and RTX in NMOSD from the perspectives of clinical and MRI activity. RTX was superior to IST in controlling NMOSD relapses and disease activity. Compared to TAC and AZA, RTX and MMF may be better treatment options NMOSD patients.

## Data Availability

The raw data supporting the conclusions of this article will be made available by the authors, without undue reservation.
