# Supplementary figures and images for: Immunotherapy for neuromyelitis optica spectrum disorder: a comparative analysis of efficacy and safety of azathioprine, mycophenolate mofetil, tacrolimus, and rituximab
Source: Front Neurol. 2025 Apr 28;16:1559118. doi: 10.3389/fneur.2025.1559118 (PMC12066257; doi:10.3389/fneur.2025.1559118)

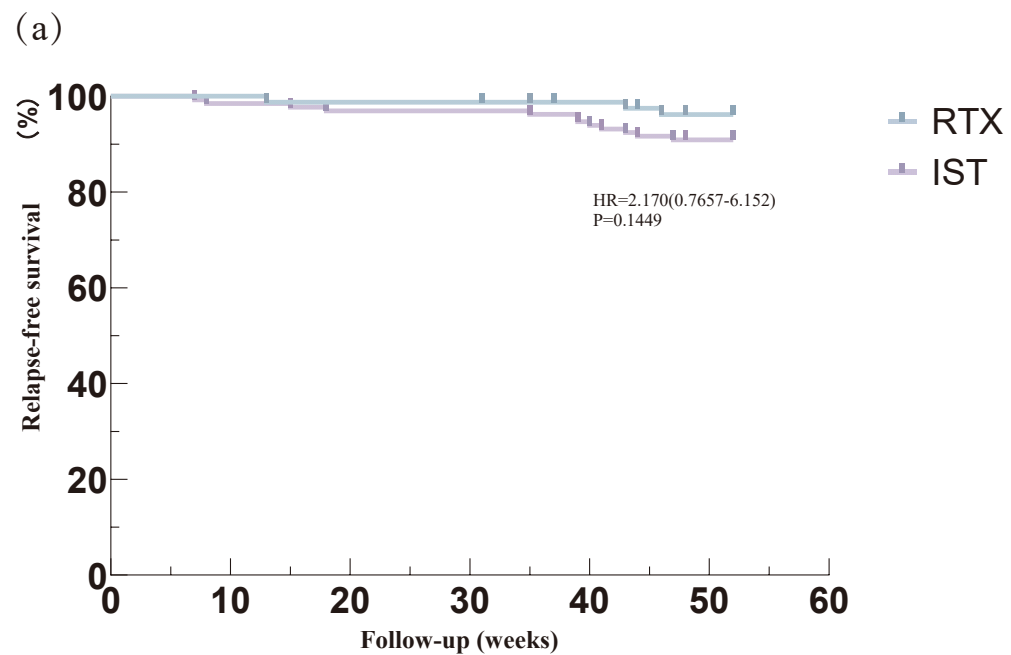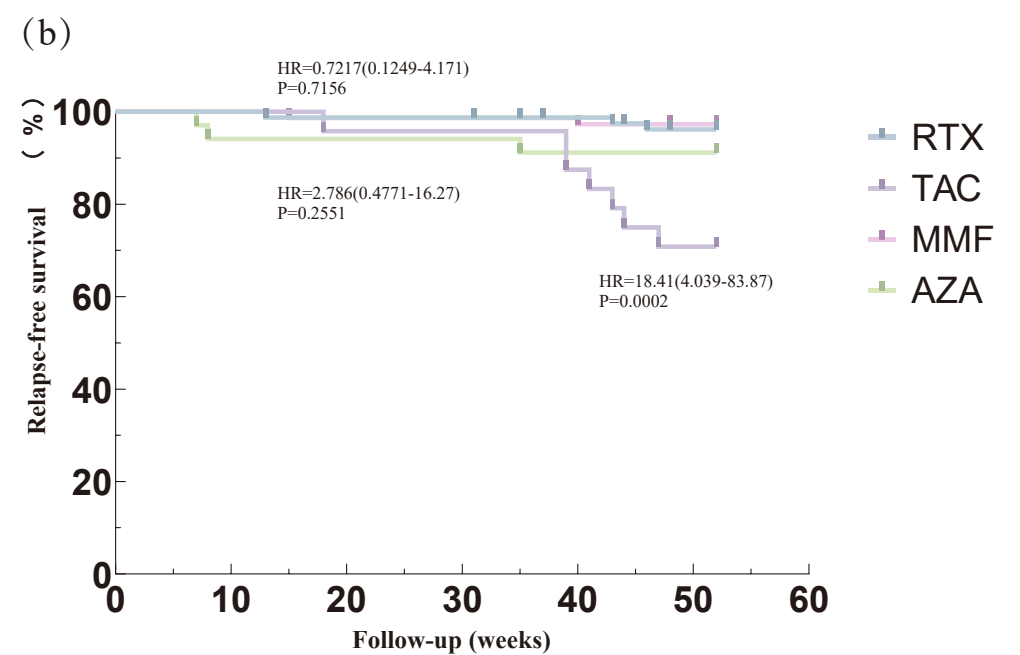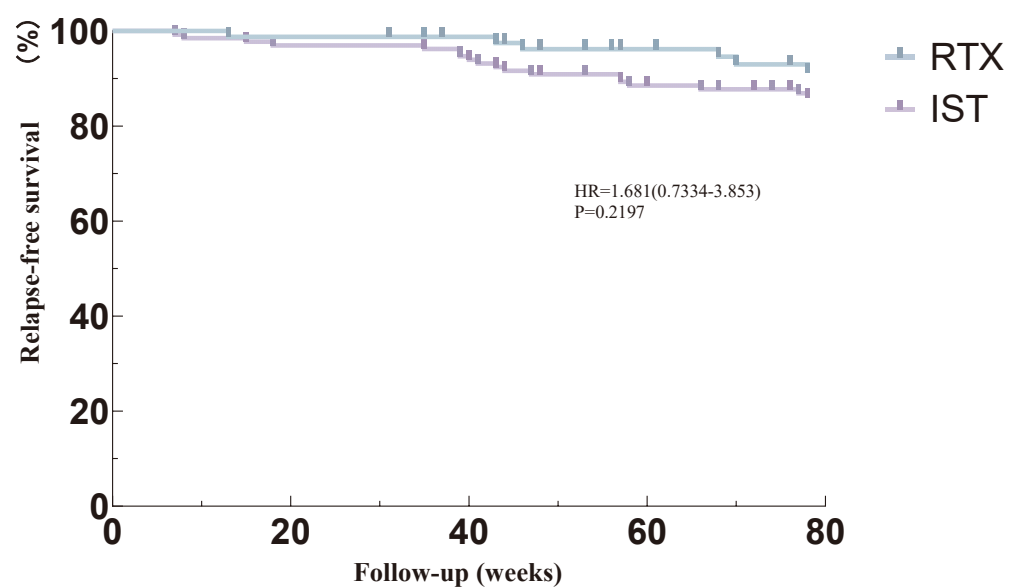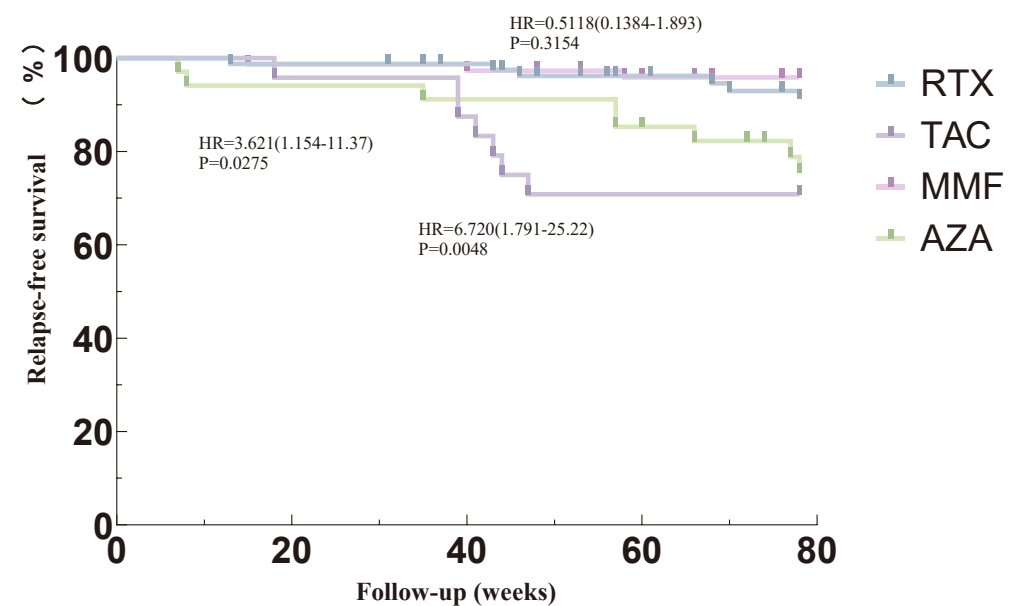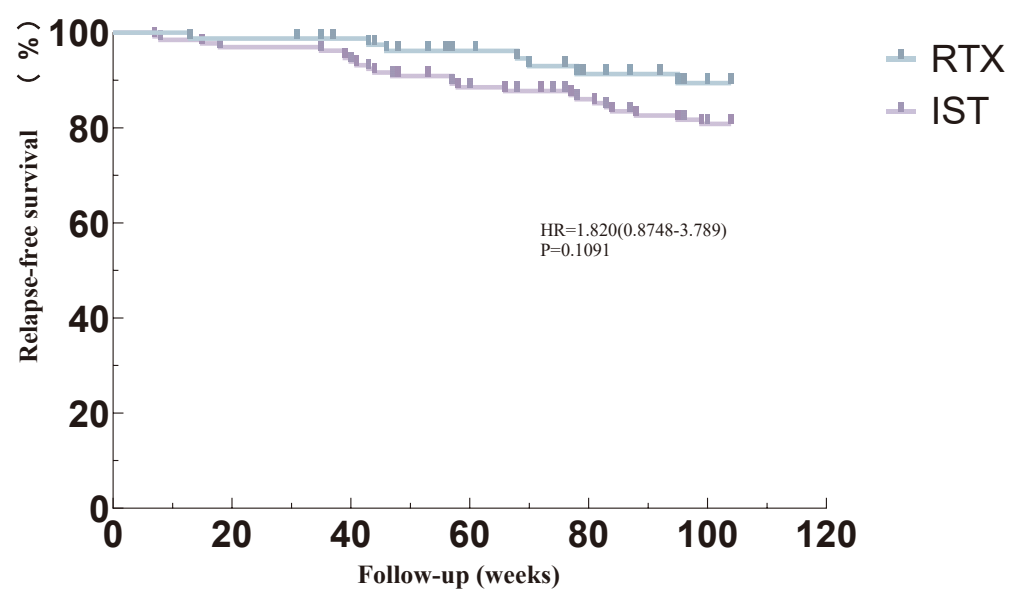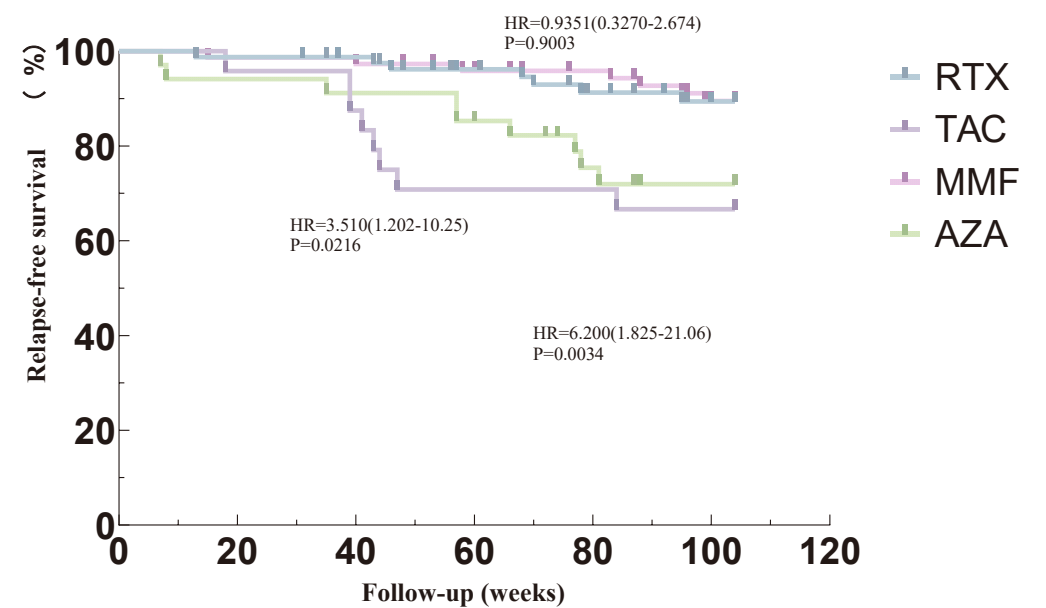

Supplement: Supplementary file 1 [file Data_Sheet_1.pdf]
